# Shenhuang granule in the treatment of severe coronavirus disease 2019 (COVID-19): study protocol for an open-label randomized controlled clinical trial

**DOI:** 10.1186/s13063-020-04498-6

**Published:** 2020-06-24

**Authors:** Bangjiang Fang, Wen Zhang, Xinxin Wu, Tingrong Huang, Huacheng Li, You Zheng, Jinhua Che, Shuting Sun, Chao Jiang, Shuang Zhou, Jun Feng

**Affiliations:** 1 Department of Emergency, LongHua Hospital, Shanghai University of Traditional Chinese Medicine, NO.725 Wanping South Road, Xuhui District, Shanghai, 200032 China; 2 Clinical Medical College of TCM, Hubei University of Chinese Medicine, NO.1 Tanhualin, Wuchang District, Wuhan, 430065 Hubei China; 3 Huangshi Hospital of TCM (Infectious Disease Hospital), NO.6 Plaza Road, Huangshi Port District, Huangshi, 435000 Hubei China; 4 The Third Department of Neurology, The Second Affiliated Hospital of Xi’an Medical University, NO.167, Textile City East Street, Baqiao District, Xi’an, 710032 Shanxi China; 5 Shanghai University of Traditional Chinese Medicine, 1200 Cai Lun Road, Zhangjiang Hi-Tech Park, Pudong New Area, Shanghai, 201203 China; 6 Department of Emergency Internal Medicine, Tongji Hospital, Tongji Medical College, Huazhong University of Science and Technology, No.1095 Jie Fang Avenue, Hankou, Wuhan, 430030 Hubei China

**Keywords:** Severe COVID-19, Shenhuang granule, “Truncation and Reversion” strategy, Randomized controlled trial

## Abstract

**Background:**

Currently, coronavirus disease 2019 (COVID-19) is continuously and rapidly circulating, causing heavy damage on public health. No effective antiviral treatment has been proved thus far. Traditional Chinese medicine (TCM) has been widely applied in the treatment of a variety of infection diseases in China, hoping to produce clinical effects and reduce the use of antibiotics and glucocorticoid. The aim of this study is to evaluate the efficacy and safety of Shenhuang granule in treatment of severe COVID-19.

**Methods/design:**

This multicenter, open-label randomized controlled trial is conducted in 160 participants with severe COVID-19. The participants will be randomly (1:1) divided into treatment group or control group. All participants are given standard therapy at the same time. The experiment will receive Shenhuang granule treatment twice a day for 14 days. The clinical indicators of patients will be assessed at baseline and at 3, 5, 7, and 14 days after treatment initiation. The primary outcome is 14-day clinical outcome. Adverse events will be monitored throughout the trial.

**Discussion:**

This will be the first randomized controlled trial, which evaluate the effect of Shenhuang granule in patients with severe COVID-19 in China. The results of this trial may not only provide evidence-based recommendations to clinicians to treat severe COVID-19, but also enrich the theory and practice of TCM in treating infectious diseases.

**Trial registration:**

Chinese Clinical Trial Registry, ChiCTR2000029777. Registered on 13 February 2020.

## Introduction

In December 2019, an outbreak of unexplained pneumonia has been diagnosed in Wuhan, Hubei Province, in China, with an epidemiological link to seafood and wet animal wholesale market where there was also sale of live animals [1, 2]. The Chinese scientists isolated an unknown coronavirus from the patient’s respiratory epithelial cells of patients and performed genome sequencing of the virus [3]. Now, this new virus is named “severe acute respiratory syndrome coronavirus 2 (SARS-CoV-2),” and it causes “coronavirus disease 2019 (COVID-19).” Although COVID-19 is considered to be the origin of zoonotic diseases, human-to human transmission has been occurred [4]. It initially erupted in Wuhan and later spread to all parts of the world, resulting in 2,954,222 confirmed cases, including 202,597 deaths globally as 28 April 2020, which caused a global health emergency [5].

The COVID-19 belongs to the family Coronaviridae and order Nidovirales, and is detected in bats, with similarity in clinical features and genetic sequence to SARS-CoV [3, 6]. Many patients of COVID-19 suffer from fever, fatigue, cough, chest tightness, and dyspnea and some progress to sepsis or septic shock or died of the infection [7]. The diagnosis of new cases includes clinical characteristics, the epidemiological history, and laboratory test according to the “Guidelines for the Diagnosis and Treatment of Novel Coronavirus (COVID-19) Infection by the National Health Commission (Trial Version 7)” [8]. Currently, there is no vaccine or specific antiviral drugs for the infection except meticulous supportive care [9].

TCM, as an important part of complementary and alternative medicine, has been widely applied in preventing and treatment of epidemic disease in China for hundreds of years. Patients with severe COVID-19 often develop into sepsis or septic shock [7]. Severe COVID-19 belongs to the “epidemic febrile disease” in TCM, and the basic pathogenesis is pathogenic toxin and blood stasis. Additionally, severe COVID-19 has some characters of “the acute deficiency syndrome.” As an innovation theory, it refers to the pathological state with a rapid consumption of the vital Qi in the human body, caused by acute and severe pathological factors such as plague, poisoning, hemorrhage, loss of fluid, and all kinds of trauma [10, 11]. Based on TCM characteristic theory “six hollow organs must keep its unobstructed” and “acute deficiency syndrome,” our team put forward “Truncation and Reversion” strategy [12], and the principle on TCM for emergency, “Treating the primary (ben) for emergency cases” [10], timely controlling the course of disease and reverse disease. On the foundation of the above theories, we developed the Shenhuang granule, a formula which is composed of six medicinal herbs, including *Panax ginseng* (*Renshen*), *Rheum palmatum L.* stem (*Dahuang*), *Sargentodoxa cuneate* (*Hongteng*), *Taraxacum mongolicum* (*Pugongying*), *Aconiti Lateralis Radix Praeparata* (*Fuzi*), and Whitmania pigra Whitman (*Shuizhi*). Rhubarb is considered a purgative and bactericidal agent to reduce heat and promote blood circulation and resolve dampness [13]. *Sargentodoxa cuneate* and *Taraxacum mongolicum* have the same function of clearing heat and detoxifying, activating blood, and removing wind and relieving pain [14, 15]. Our previous studies have demonstrated that Jinhong Decoction, composed of *Rheum palmatum L*. stem, *Sargentodoxa cuneata*, and *Taraxacum mongolicum*, can inhibit the levels of TNF-α, IL-6, IL-8, and other inflammatory cytokines, protect against excessive inflammatory response, and maintain organism’s balance between inflammation and anti-inflammatory in severe infectious diseases [16, 17]. Ginseng has the functions to strongly tonify the Qi, nourishing lungs and spleen, promoting the secretion of saliva and quenching thirst, strengthening the heart, and calming the mind [18]. Several studies recently report that ginseng can directly kill bacteria and regulate bacterial adhesion, inflammation, cytotoxicity, and hemagglutination [19]. Wang et al. [20] report that ginseng extracts can inhibit different strains of influenza viruses. *Aconiti Lateralis Radix Praeparata* is considered an effective stimulant for the spleen and kidneys [21]. Recent studies report that aconite had antibacterial activity, especially against *S*. *aureus* and *E*. *coli*, as well as immunoregulation efficacy [22, 23]. Whitmania pigra Whitman has functions of breaking stagnant and eliminating blood stasis. Yao et al. [24] believe that Whitmania pigra Whitman extract effectively reduces deep vein thrombosis by inhibiting inflammation via modulating silent information regulator 1 (SIRT1)/nuclear factor-κB (NF-κB) signaling pathway. Therefore, according to the theory of TCM and modern Western medical research, Shenhuang granule should have beneficial and curative effects on severe COVID-19 [11, 12, 25].

We design this study as a multicenter, randomized, and controlled clinical trial based on the “Truncation and Reversion” strategy of TCM innovation theory and “acute deficiency syndrome.” The aim of this clinical trial is to confirm the effectiveness and safety of Shenhuang granule in blocking the deterioration of severe COVID-19 and provide reliable evidence for clinical research.

## Methods/design

### Study design and settings

**Fig. 1:**
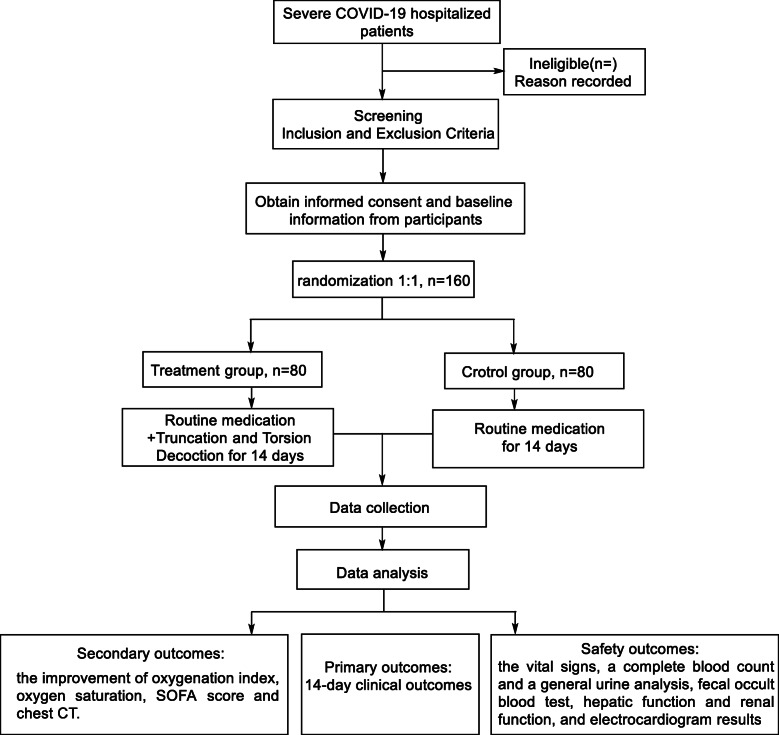
Flow chart of the study design. COVID-19, coronavirus disease 2019; SOFA score, Sequential Organ Failure Assessment score; CT, computed tomography

The study will be a randomized, controlled, multicenter trial conducted at 4 medical centers, selected by the coordination center. The coordinating center comprise clinical experts, statisticians, and quality control experts, who will be responsible for clinical research methodology and resolving the key issues in the progress of research implementation. And they also have the responsibilities, including the design of the clinical research trial, selection of the cooperative hospitals, and provision of training courses including instructions manuals. The medical centers enrolled in this study are the Huangshi Hospital of Traditional Chinese Medicine, Tongji Hospital Tongji Medical College Huazhong University of Science and Technology, LaoHeKou Tradiational Chinese Medicine Hospital, and Leishenshan Hospital of Wuhan. A total of 160 patients that meet the eligibility criteria will be randomized into two groups (treatment group and control group) at a ratio of 1:1. The study flowchart is illustrated in Fig. 1. The Standard Protocol Items Recommendations for Interventional Trials (SPIRIT) checklist is presented in Additional file 1.

### Objectives

The primary objectives of this trial are to evaluate the efficacy and safety of Shenhuang granule in patients with severe COVID-19.

### Population

Participant recruitment takes place daily by the study personnel from the 4 study sites in China. Subjects who meet the inclusion criteria and sign the informed consent forms will enter the screening period. Patients who meet the exclusion criteria will be excluded before randomization. The recruitment duration will last for 4 months, from February 2020 to June 2020.

### Inclusion criteria

Subjects must meet all the following requirements:

1. Hospitalized patients with 2019 novel coronavirus confirmed by SARS-CoV-2 nucleic acid testing of the respiratory specimens which showed positive results by means of real-time reverse transcription polymerase chain reaction (RT-PCR) assay.
2. Meet any one of the criteria for severe type:
3. Respiratory distress: RR ≥ 30/min
4. Blood oxygen saturation of resting state ≤ 93%
5. Arterial oxygen partial pressure (PaO_2_)/fraction of inspiration O2(FiO_2_)(P/F) ≤ 300 mmHg
6. Be 18–80 years of age
7. Provide signed informed consent form

### Exclusion criteria

The exclusion criteria are as follows:

1. Pregnant and lactating women
2. Allergic to one or more substance in “Shenhuang granule”
3. Severe primary disease including malignant tumor, blood diseases, HIV, and severe liver and kidney dysfunctions
4. Obstructive pneumonia, interstitial pulmonary fibrosis, alveolar proteinosis, and hypersensitivity pneumonitis caused by obstructive lung tumors

### Patient withdrawal

The rejection and withdrawal criteria are as follows:

1. Adverse events and complications occur, and the investigator considers that it is necessary to terminate the trial
2. Voluntary withdrawal
3. Those who are lost follow-up

In patient withdrawal, all study interventions will cease. All data captured up to this point in time is stored in the database, and safety-related data are documented until day 14 if possible. Data from such patients become the dataset for efficacy and safety analysis on day 14.

### Monitoring of compliance

The methods to improve the compliance of researchers mainly include the following: (1) selecting researchers carefully and rigorously, (2) strict and comprehensive training and assessment, (3) streamlining the research process, (4) strengthen communication and supervision, and (5) appropriate incentives. In this study, the subjects are seriously ill. There is no specific medicine for this disease, and the patients have relatively high compliance with TCM.

However, the following aspects still need to be strengthened by the investigator’s control of the subject’s compliance: (1) the researcher who talks with the patient or their legal agents for informed consent should have strong communication skills and rich clinical experience, be able to communicate with empathy, and answer patients’ questions to obtain full understanding and trust, so as to avoid non-compliance after enrollment and (2) subjects should maintain necessary contact after being transferred or discharged from the hospital after the end of the drug treatment period.

### Sample size

A previous study [26] on COVID-19 by traditional Chinese medicine showed that the clinical cure rate in the conventional treatment group was 61.11%, while it was 91.18% in the group receiving traditional Chinese medicine plus routine treatment. According to the literature, presuming that the rate of the clinical effective rate in the treatment group is improved from 61.11 to 91.18%, the sample size is calculated according to the parameters *α* = 0.05 (two-sided test) and *β* = 0.1. Comparing the rates of the two groups with respect to the sample size estimation formula, we calculated that 41 patients should be recruited in each group. Studies with a similar patient population have the drop-out rate of 10% [27], and we will enroll a total sample size of 160 participants. We used the calculation tool on https://www.cnstat.org/samplesize/.

### Randomization, allocation concealment, and blinding

The randomization sequence is generated by SPSS 19.0, using permuted small blocks of random size, stratified by medical center, and grouping the Chinese medicine group and the control group on a 1:1 schedule. The two sets of allocation codes are kept in opaque envelopes; one set is kept by the research sponsor and the other set by the pharmacy administrators, who then assign the study drug to the nurses for administration. This is an open-label study. The allocation concealment is blinded before the envelop is opened. After randomization, subjects randomized to the intervention arm will be receiving combined Shenhuang granule and Western medicine therapy, or only Western medicine therapy. The evaluators and statistician will be blinded until the completion of the visit and analysis. The statistical analysis is carried out by the professors of statistics at the Shanghai University of Traditional Chinese Medicine.

### Interventions

The Shenhuang granule comprises 50 g of *Panax ginseng C. A. Mey*, 40 g of *Rheum palmatum L.* stem, 30 g of *Sargentodoxa cuneata* stem, 30 g of *Taraxacum mongolicum*, 50 g of *Aconiti Lateralis Radix Praeparata*, and 6 g of Whitmania pigra Whitman, which are packaged into two sachets. Patients in both groups can retain standard care according to the “Guidelines for the Diagnosis and Treatment of Novel Coronavirus (COVID-19) Infection (Trial Version 7)” by the National Health Commission [8]. The treatment group will be given standard Western medicine therapy and Shenhuang granule (oral, two sachets per day) for 14 days, while the control group only receives standard Western medicine therapy for 14 days. The routine care should include early fluid resuscitation, antimicrobial anticoagulants, nutritional support, and other treatments. The Shenhuang granule is provided and manufactured by the Beijing Tcmages Pharmaceutical Co., LTD (Number: Jing 20180032). Both groups then undergo a 14-day treatment. The use of all medication information is documented in the case report form (CRF).

### Outcomes and measurements

The primary outcome is clinical improvement up to 14 days after treatment. The definition of clinical improvement is that the patient’s admission status drops by 1 point on the 6-point scale or is discharged from the hospital, whichever comes first. The 6-point ordinal scale based on the well-defined WHO severity scale is as follows: 6 = death; 5 = critical, meet any of the following conditions: (1) respiratory failure, mechanical ventilation is required, (2) shock, (3) with other organ failure which requires ICU care; 4 = severe, meet any of the following criteria: (1) respiratory distress (≥ 30 breaths/ min), (2) oxygen saturation at rest ≤ 93%, and (3) arterial partial pressure of oxygen (PaO_2_)/fraction of inspired oxygen (FiO_2_) ≦ 300 mmHg; 3 = moderate, fever and respiratory symptoms, accompanied by radiological findings of pneumonia; 2 = mild, mild clinical symptoms, and no imaging signs of pneumonia; and 1 = discharged alive, (1) body temperature returned to normal for more than 3 days, (2) respiratory symptoms improved markedly, (3) pulmonary imaging showed obvious absorption of inflammation, and (4) the nucleic acid test of respiratory tract specimen is negative for 2 consecutive times.

Secondary outcomes are the mortality and rate of receiving invasive ventilator in the severe category, the improvement of oxygen saturation, and Acute Physiology and Chronic Health Evaluation (APACHE-II) score.

### Safety outcomes

At baseline and at each study visit, safety checks will be performed on the patients by evaluating the vital signs, complete blood count and general urine analysis, fecal occult blood test, hepatic function and renal function, and electrocardiogram results. Any adverse event that occurs during the study are observed and recorded in detail.

### Adverse event reporting

An adverse event (AE) refers to any syndrome or disease that appears or worsens during the study. The investigators record any AE in the AE form timely, including occurrence time, severity, duration, the measures taken, and the outcome. Relevant information should be submitted to the sponsors, ethics committees, and drug supervision and administration department in accordance with the regulation. The incidences of serious AEs in patients are promptly reported to the principal investigator and the adverse drug reactions to the monitoring center of the local authority within 24 h.

### Statistical analysis

No interim analysis or subgroup analyses are planned for this trial. Full analysis set (FAS), per-protocol set (PPS), and safety analysis set (SS) will be conducted. FAS refers to the ideal set of eliminating of all subjects in a minimum and reasonable manner, according to the principle of intention-to-treat (ITT). PPS is a subset of ITT’s dataset, which has completed relevant observation according to the program requirements. The compliance of these subjects ranges from 80 to 120%, in which the subjects do not seriously violate the research program, complete all visits and CRF filling, and do not miss the main evaluation indexes and most other evaluation indexes. SS refers to all subjects who receive at least one treatment after randomization. All safety information records of subjects will be evaluated, including adverse events, severe adverse events, and clinically significant changes in safety indicators. The statistical analysis is performed in a blinded manner using the SPSS 20.0 software. For the continuous variables, the chi-squared test or Fisher’s exact test is used to compare the clinical improvement, oxygen saturation, and Acute Physiology and Chronic Health Evaluation (APACHE-II) score. Relative risk, risk difference, and number of treatments are reported with a 95% confidence interval. For the two categorical variables, we make comparison between groups and calculate the 95% CI using chi-squared tests. For the repeated measures data, category variance outcomes are analyzed using generalized estimating equations, and conclusions are drawn according to the estimating of parameter and standard error.

Our approach is to use the final observation status when no mortality is observed for 14 days, to ensure that we do not lose data about the primary outcome and the key secondary outcome. A *P* value < 0.05 is considered as statistically significant.

### Trial management

#### Coordination center

It is composed of clinical experts, statisticians, and quality control experts from the LongHua Hospital, Shanghai University of Traditional Chinese Medicine, and Huangshi Hospital of TCM. The center is responsible for clinical research methodology, resolving key issues in the implementation of the study, designing clinical research trials, selecting cooperative hospitals, and providing training courses including instruction manual.

#### Quality control

Sites and researchers are monitored and inspected regularly by Clinical Research Organizations (CRO), following the standard protocol throughout the process.

### Data collection and management

Trial data are carefully collected by the research team in accordance with a standard protocol and then paper CRFs will be completed in an accurate, timely, and reliable manner. The data is input it into an Electronical Data Capture system and is regulated and checked by the monitor. The modifications made by investigators should be checked promptly, and the feedback is provided to the researchers and the monitor. All CRF papers transmitted between investigators, inspectors, and data managers should be documented and kept properly. The data management locks the data once the study is completed. The medical information of all study participants will be kept strictly confidential. The SPIRIT flowchart of the trial is shown in Table 1. We will disseminate data and research findings via publication.

**Table 1:**
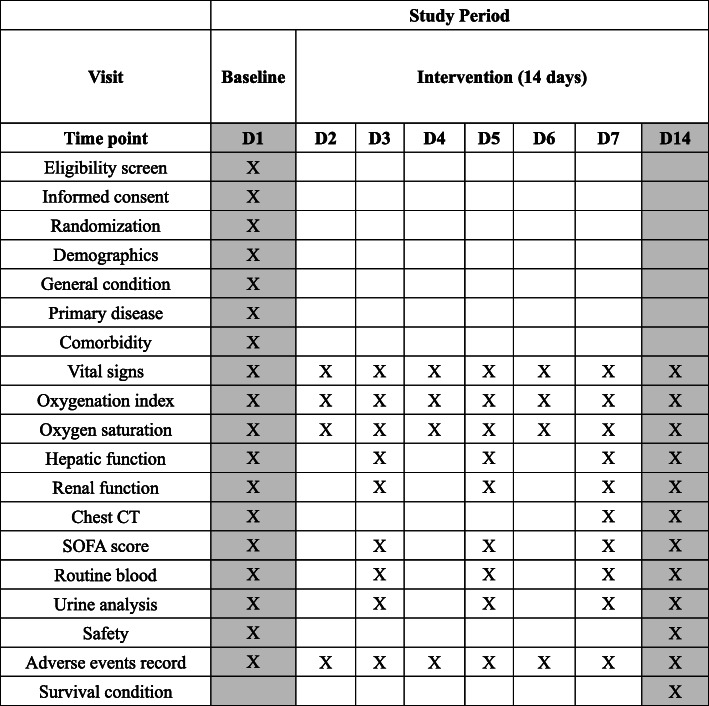
Study procedures and assessments

*CT* computed tomography, *SOFA score* Sequential Organ Failure Assessment score

### Quality control

All the staff involved the trial should be qualified in the implementation of Good Clinical Practice (GCP) training by the State Food and Drug Administration to ensure quality of training. Before the trial, the investigators should have undergone rigorous training and taken comprehensive tests to understand the details of the trial and improve compliance. Qualified researchers should collect the accuracy and completeness of clinical data to complete the CRF. Monitoring is performed by Tianjin jinshi kangsheng pharmaceutical technology co. LTD in Tianjin once a month. All monitoring procedures, such as frequency of visits, and source data verification follow the standard protocol throughout the process. All information recorded on case report forms must be traceable to the original file in the patient’s file; the original documents are kept.

### Ethics

Study participants or their legal representatives have obtained sufficient explanation and time to sign the informed consent form before the trial. Researchers ensure that the study is conducted in accordance with the principles of Declaration of Helsinki and the clinical trial quality management regulations in China. The study protocol has been approved by the Ethics Committee of Huangshi Hospital of Traditional Chinese Medicine (approval number HSZY-PJ-2020-001-01) and registered with the Chinese Clinical Trial Registry (ChiCTR2000029777). All breaches of confidentiality, changes of study protocol, or AEs in this study will be retained as a program addendum and submitted to the Ethics Committee for re-review.

## Discussion

COVID-19 mainly causes respiratory tract infections, high infectivity and fatality rate, and serious harm on public health [2]. Common clinical presentations are fever, fatigue, and dry cough and some are accompanied by diarrhea [3]. Several severe patients presented with dyspnea, and some rapidly developed sepsis or septic shock [3]. It is an urgent issue to develop effective drugs against the virus. From the perspective of TCM, pathogenic toxin, blood stasis, and Qi deficiency are considered the main causes of severe COVID-19. According to the innovation theory of “Truncation and Reversion” strategy and “acute deficiency syndrome,” our team developed the Shenhuang granule, a Chinese medicine compound for the symptomatic treatment of severe COVID-19. Shenhuang granule contains *Panax ginseng*, *Rheum palmatum L.* stem, *Sargentodoxa cuneata*, *Taraxacum mongolicum*, *Aconiti Lateralis Radix Praeparata*, and Whitmania pigra Whitman. The TCM compounds have antipyretic and purgative effects, promoting blood circulation and removing blood stasis, as well as tonifying the Qi, lung, and spleen. Preliminary clinical trial results indicated that Jinhong Decoction is composed of *Rheum palmatum L.* stem, *Sargentodoxa cuneata*, and *Taraxacum mongolicum*, which can significantly reduce mortality, mechanical ventilation time, and the use of antibiotic, with no adverse reactions such as liver and kidney dysfunction in patients with infection diseases [28, 29]. Therefore, the results of this clinical trial can provide valuable evidence on Shenhuang granule in the treatment of severe COVID-2019. However, the safety of the six medicinal herbs to be administered to patients should always be a concern. A number of preclinical and clinical studies have testified that the dosages of these herbs are far less than toxic dose and are used frequently in clinical treatment with good result, especially in treating severe and critical symptoms [30–34]. In addition, all the above herbs are qualified with the national standards.

This trial is designed as a multicenter, open-label randomized and controlled clinical study from the perspective of evidence-based medicine and is considered to be the most definitive research method of treatment evaluation of Shenhuang granule. In this trial, we select the 14-day clinical improvement as the primary outcome. The 14-day clinical improvement directly reflects the condition of severe COVID-19 patients and the efficacy of TCM treatment. The secondary outcomes of this trial include the mortality and rate of receiving invasive ventilator in the severe category, the improvement of oxygen saturation, and Acute Physiology and Chronic Health Evaluation (APACHE-II) score. The selected indicators in this study are used to reflect the condition of patients and to evaluate the effectiveness and safety of Shenhuang granule.

We hope this study could further explain the scientific connotation of the treatment of severe COVID-19 based on TCM innovation theory “Truncation and Reversion” strategy and “acute deficiency syndrome,” and open up new ideas and methods for clinical exploration of prevention strategy, TCM, and integrated medicine diagnosis and therapy of COVID-19. Nevertheless, the design of the trial also has potential limitations. The impact of some of the end points, such as a long-term mortality, has not yet been analyzed.

### Trial status

The protocol version is 1.0, 1 February 2020. We are currently recruiting participants from February 2020. It is estimated that up to 160 participants will be enrolled by June 2020.

## Supplementary information

**Additional file 1.** SPIRIT Checklist.

**Additional file 2.** Informed Consent Form.

**Additional file 3.** Inspection Report.

## Data Availability

Not applicable

## Abbreviations

COVID-2019: 2019 novel coronavirus
SOFA: Sequential Organ Failure Assessment
IL-6: Interleukin-6
TNF-α: Tumor necrosis factor
SIRT1: Silent information regulator 1
NF-κB: Nuclear factor-κB
AE: Adverse event
AEs: Adverse events
CRF: Case report form
SPIRIT: Standard Protocol Items Recommendations for Interventional Trials
CT: Chest computed tomography
GCP: Good Clinical Practice
TCM: Traditional Chinese medicine
