# Supplementary material for: Shenhuang granule in the treatment of severe coronavirus disease 2019 (COVID-19): study protocol for an open-label randomized controlled clinical trial
Source: Trials. 2020 Jun 24;21:568. doi: 10.1186/s13063-020-04498-6 (PMC7312108; doi:10.1186/s13063-020-04498-6)
Supplement: Supplementary file 3 — Additional file 3. Inspection Report. [file 13063_2020_4498_MOESM3_ESM.docx]

**Inspection Report**

File Code: SOPD1022-RD05-03

Report No.: C201909178

| Raw Material | □ | Net Material | □ | Intermediate | □ | Bulk Product | □ |
| --- | --- | --- | --- | --- | --- | --- | --- |
| Finished product | ■ | Auxiliary Material | □ | Packing Material | □ | Re-inspection | □ |

| Name | Sargentodoxa Cuneata Formula Granules | Material Code | 1000000388 |
| --- | --- | --- | --- |
| Specification | 100g/ bottle | Batch No. | 19033991 |
| Quantity (batch) | 2380 bottles | Inspection Quantity | 400g |
| Source |  | Place of Origin | Fengcheng, Yichun City, Jiangxi Province |
| Test Date | 2019-09-11 | Inspection Item | Full inspection |
| Issue Date | 2019-09-29 | Inspection No. | C1909178 |
| Inspection Criteria | "Quality Standard of Sargentodoxa Cuneata Formula Granule", "Standard Operating Procedure for Inspection of Sargentodoxa Cuneata Formula Granule" | | |

| Appearance | yellowish brown to reddish brown granules; slight smell, bitter taste, mild medicinal property | Compliance |
| --- | --- | --- |
| Identification |  |  |
| TLC | The position and color of the main spot of the test product should be the same as that of the Sargentodoxa Cuneata. | Compliance |
| Inspection |  |  |
| Moisture Content | Should not exceed 8.0% | 4.02% |
| Particle Size | Should not exceed 13% | 4.7% |
| Solubility | Should be completely dissolved | Compliance |
| Quantity | Should meet the requirements | Compliance |
| Extractum | Should not be less than 26.0% | 36.2% |
| Infrared Fingerprint | conform to the Atlas of Sargentodoxa Cuneata Formula Granules | Compliance |
| Microbial Limit 1 | total number of aerobic bacteria not exceed 2000cfu/g | <10cfu/g |
| Microbial Limit 2 | total number of molds and yeasts not exceed 200cfu / g | <10cfu/g |
| Control Bacteria Examination | Escherichia coli must not be detected / g | Not detected / g |

**Conclusion:**

The above items are inspected according to the "Quality Standard of Sargentodoxa Cuneata Formula Granules", and the results conform to the regulation.

Editor: Jiamei Wang Reviewer: Xiu Sun Approver: Jun An Official Seal of Quality Inspection

(Beijing Tcmages Pharmaceutical Co., Ltd. Quality Inspection Special Seal)

**Inspection Report**

File Code: SOPD1022-RD05-03

Report No.: C201909387

| Raw Material | □ | Net Material | □ | Intermediate | □ | Bulk Product | □ |
| --- | --- | --- | --- | --- | --- | --- | --- |
| Finished product | ■ | Auxiliary Material | □ | Packing Material | □ | Re-inspection | □ |

| Name | Heishun Tablet Formula Granules | Material Code | 1000000052 |
| --- | --- | --- | --- |
| Specification | 100g/ bottle | Batch No. | 19034081 |
| Quantity (batch) | 7567 bottles | Inspection Quantity | 400g |
| Source |  | Place of Origin | Cangxi, Guangyuan City, Shichuan |
| Test Date | 2019-09-24 | Inspection Item | Full inspection |
| Issue Date | 2019-09-30 | Inspection No. | C1909387 |
| Inspection Criteria | "Quality Standard of Heishun Tablet Formula Granules", "Standard Operating Procedure for Inspection of Heishun Tablet Formula Granules" | | |

| Appearance | Light yellow to yellowish-brown granules; slight smell, light taste | Compliance |
| --- | --- | --- |
| Identification |  |  |
| TLC | The location and color of the main spot of the test product should be the same as that of the benzoyl aconitine reference products. | Compliance |
| Inspection |  |  |
| Moisture Content | Should not exceed 8.0% | 4.65% |
| Particle Size | Should not exceed 13% | 2.9% |
| Solubility | Should be completely dissolved | Compliance |
| Quantity | Should meet the requirements | Compliance |
| Extractum | Should not be less than 15.0% | 25.1% |
| Infrared Fingerprint | conform to the Atlas of Heishun Tablet Formula Granules | Compliance |
| Microbial Limit 1 | total number of aerobic bacteria not exceed 2000cfu/g | <10cfu/g |
| Microbial Limit 2 | total number of molds and yeasts not exceed 200cfu / g | <10cfu/g |
| Control Bacteria Examination | Escherichia coli must not be detected / g | Not detected / g |

**Conclusion:**

The above items are inspected according to the "Quality Standard of Heishun Tablet Formula Granules", and the results conform to the regulation.

Editor: Jiamei Wang Reviewer: Xiu Sun Approver: Jun An Official Seal of Quality Inspection

(Beijing Tcmages Pharmaceutical Co., Ltd. Quality Inspection Special Seal)

**Inspection Report**

File Code: SOPD1022-RD05-03

Report No.: C201912715

| Raw Material | □ | Net Material | □ | Intermediate | □ | Bulk Product | □ |
| --- | --- | --- | --- | --- | --- | --- | --- |
| Finished product | ■ | Auxiliary Material | □ | Packing Material | □ | Re-inspection | □ |

| Name | Dandelion Formula Granules | Material Code | 1000000512 |
| --- | --- | --- | --- |
| Specification | 250g/ bottle | Batch No. | 19047721 |
| Quantity (batch) | 3427 bottles | Inspection Quantity | 1000g |
| Source |  | Place of Origin | Longxi, Dingxi City, Gansu Province |
| Test Date | 2019-12-28 | Inspection Item | Full inspection |
| Issue Date | 2020-01-02 | Inspection No. | C1912715 |
| Inspection Criteria | "Quality Standard for Dandelion Formula Granules", "Standard Operating Procedures for Inspection of Dandelion Formula Granules" | | |

| Appearance | Light brownish yellow to yellow brown granules, with slight smell, bitter taste | Compliance |
| --- | --- | --- |
| Identification |  |  |
| TLC | The location and color of the main spot of the test product should be the same as that of the Dandelion control medicinal material and the caffeic acid control products. | Compliance |
| Inspection |  |  |
| Moisture Content | Should not exceed 8.0% | 6.00% |
| Particle Size | Should not exceed 13% | 3.2% |
| Solubility | Should be completely dissolved | Compliance |
| Quantity | Should meet the requirements | Compliance |
| Content Determination | Calculated as a dry product and contain no less than 0.4mg of caffeic acid per 1g | 0.8mg/g |
| Extractum | Should not be less than 20.0% | 26.5% |
| Infrared Fingerprint | conform to the Atlas of Dandelion Formula Granules | Compliance |
| Microbial Limit 1 | total number of aerobic bacteria not exceed 2000cfu/g | <10cfu/g |
| Microbial Limit 2 | total number of molds and yeasts not exceed 200cfu / g | 10cfu/g |
| Control Bacteria Examination | Escherichia coli must not be detected / g | Not detected / g |

**Conclusion:**

The above items are inspected according to the "Quality Standard of Dandelion Formula Granules", and the results conform to the regulation.

Editor: Xueting Li Reviewer: Xiu Sun Approver: zhaofu Liu Official Seal of Quality Inspection

(Beijing Tcmages Pharmaceutical Co., Ltd. Quality Inspection Special Seal)

**Inspection Report**

File Code: SOPD1022-RD05-03

Report No.: C201912006

| Raw Material | □ | Net Material | □ | Intermediate | □ | Bulk Product | □ |
| --- | --- | --- | --- | --- | --- | --- | --- |
| Finished product | ■ | Auxiliary Material | □ | Packing Material | □ | Re-inspection | □ |

| Name | Ginseng Formula Granules | Material Code | 1000000091 |
| --- | --- | --- | --- |
| Specification | 100g/ bottle | Batch No. | 19042681 |
| Quantity (batch) | 6517 bottles | Inspection Quantity | 400g |
| Source |  | Place of Origin | Fusong, Baishan City, Jilin Province |
| Test Date | 2019-11-30 | Inspection Item | Full inspection |
| Issue Date | 2020-12-06 | Inspection No. | C1912006 |
| Inspection Criteria | "Ginseng Formula Granule Quality Standard", "Standard Operating Procedures for Inspection of Ginseng Formula Granule" | | |

| Appearance | white to yellow-white granules; slight smell, slightly sweet and bitter taste | Compliance |
| --- | --- | --- |
| Identification |  |  |
| TLC | The location and color of the main spot of the test product should be the same as that of the ginseng reference medicinal materials, and reference products of Ginsenoside Rb1, Ginsenoside Re, Ginsenoside Rg1 and Ginsenoside Rf. | Compliance |
| Inspection |  |  |
| Moisture Content | Should not exceed 6.0% | 4.6% |
| Particle Size | Should not exceed 13% | 3.9% |
| Solubility | Should be completely dissolved | Compliance |
| Quantity | Should meet the requirements | Compliance |
| Content Determination 1 | calculated as a dry product, total amount of Ginsenoside Rg1 and Ginsenoside Re should not be less than 0.24% | 0.51% |
| Content Determination 2 | calculated as a dry product, total amount of Ginsenoside Rb1 should not be less than 0.20% | 0.69% |
| Infrared Fingerprint | conform to the Atlas of Ginseng Formula Granules | Compliance |
| Microbial Limit 1 | total number of aerobic bacteria not exceed 2000cfu/g | <10cfu/g |
| Microbial Limit 2 | total number of molds and yeasts not exceed 200cfu / g | <10cfu/g |
| Control Bacteria Examination | Escherichia coli must not be detected / g | Not detected / g |

**Conclusion:**

The above items are inspected according to the "Quality Standard of Ginseng Formula Granules", and the results conform to the regulation.

Editor: Wei Liu Reviewer: Xiu Sun Approver: zhaofu Liu Official Seal of Quality Inspection

(Beijing Tcmages Pharmaceutical Co., Ltd. Quality Inspection Special Seal)

**Inspection Report**

File Code: SOPD1022-RD05-03

Report No.: C201909132

| Raw Material | □ | Net Material | □ | Intermediate | □ | Bulk Product | □ |
| --- | --- | --- | --- | --- | --- | --- | --- |
| Finished product | ■ | Auxiliary Material | □ | Packing Material | □ | Re-inspection | □ |

| Name | Cooked Rhubarb Formula Granules | Material Code | 10000000444 |
| --- | --- | --- | --- |
| Specification | 250g/ bottle | Batch No. | 19033471 |
| Quantity (batch) | 2134 bottles | Inspection Q’TY | 1000g |
| Source |  | Place of Origin | Wangcang, Guangyuan City, Shichuan |
| Test Date | 2019-09-08 | Inspection Item | Full inspection |
| Issue Date | 2019-09-16 | Inspection No. | C1909132 |
| Inspection Criteria | "Cooked Rhubarb Formula Granules Quality Standard", "Standard Operating Procedures for Inspection of Cooked Rhubarb Formula Granules" | | |

| Appearance | Brown-yellow to brown granules; fresh smell, bitter and slightly astringent taste | Compliance |
| --- | --- | --- |
| Identification |  |  |
| TLC | The location and color of the main spot of the test product should be consistent with that of the reference products of aloe-emodin, rhein, emodin, chrysophanol and physcion, and rhubarb reference medicinal materials. | Compliance |
| Inspection |  |  |
| Moisture Content | Should not exceed 8.0% | 6.91% |
| Particle Size | Should not exceed 13% | 4.2% |
| Solubility | Should be completely dissolved | Compliance |
| Quantity | Should meet the requirements | Compliance |
| Rhapontin | Should not be detected | Compliance |
| Content Determination | calculated as a dry product, total amount of aloe-emodin, rhein, emodin, chrysophanol and physcion should not be less than 1.0mg per 1g | 10mg/g |
| Infrared Fingerprint | conform to the Atlas of Cooked Rhubarb Formula Granules | Compliance |
| Microbial Limit 1 | total number of aerobic bacteria not exceed 2000cfu/g | <10cfu/g |
| Microbial Limit 2 | total number of molds and yeasts not exceed 200cfu / g | <10cfu/g |
| Control Bacteria Examination | Escherichia coli must not be detected / g | Not detected / g |

**Conclusion:**

The above items are inspected according to the "Quality Standard of Cooked Rhubarb Formula Granules", and the results conform to the regulation.

Editor: Wei Liu Reviewer: Xiu Sun Approver: Jun An Official Seal of Quality Inspection

(Beijing Tcmages Pharmaceutical Co., Ltd. Quality Inspection Special Seal)

**Inspection Report**

File Code: SOPD1022-RD05-03

Report No.: C201908114

| Raw Material | □ | Net Material | □ | Intermediate | □ | Bulk Product | □ |
| --- | --- | --- | --- | --- | --- | --- | --- |
| Finished product | ■ | Auxiliary Material | □ | Packing Material | □ | Re-inspection | □ |

| Name | Water-boiled Leech Formula Granules | Material Code | 1000000573 |
| --- | --- | --- | --- |
| Specification | 100g/ bottle | Batch No. | 19026861 |
| Quantity (batch) | 1450 bottles | Inspection Quantity | 400g |
| Source |  | Place of Origin | Sishui, Jining city, Shandong |
| Test Date | 2019-08-06 | Inspection Item | Full inspection |
| Issue Date | 2019-08-13 | Inspection No. | C1908114 |
| Inspection Criteria | "Water-boiled Leech Formula Granules Quality Standard", "Standard Operating Procedures for Inspection of Water-boiled Leech Formula Granules" | | |

| Appearance | yellow-white to yellow granules; slightly fishy smell, salty taste | Compliance |
| --- | --- | --- |
| Identification |  |  |
| TLC | The location and color of the main spot of the test product should be the same as that of the leech reference medicinal materials. | Compliance |
| Inspection |  |  |
| Moisture Content | Should not exceed 8.0% | 4.61% |
| Particle Size | Should not exceed 13% | 1.8% |
| Solubility | Should be completely dissolved | Compliance |
| Quantity | Should meet the requirements | Compliance |
| Extractum | Should not be less than 5.0% | 19% |
| Infrared Fingerprint | conform to the Atlas of Water-boiled Leech Formula Granules | Compliance |
| Microbial Limit 1 | total number of aerobic bacteria not exceed 2000cfu/g | 90cfu/g |
| Microbial Limit 2 | total number of molds and yeasts not exceed 200cfu / g | <10cfu/g |
| Control Bacteria Examination 1 | Escherichia coli should not be detected / g | Not detected /g |
| Control Bacteria Examination 2 | Salmonella should not be detected / 10g | Not detected /10g |

**Conclusion:**

The above items are inspected according to the "Quality Standard of Water-boiled Leech Formula Granules", and the results conform to the regulation.

Editor: Wei Liu Reviewer: Xiu Sun Approver: Jun An Official Seal of Quality Inspection

(Beijing Tcmages Pharmaceutical Co., Ltd. Quality Inspection Special Seal)
